# Reduced circulating mitochondrial DNA integrity and increased DNA oxidation in preclinical and clinical pediatric obesity: an observational study

**DOI:** 10.3389/fped.2026.1813689

**Published:** 2026-06-29

**Authors:** Mónica M. Velásquez-Esparza, Perla Pérez-Treviño, Leticia Elizondo-Montemayor, Elena Cristina Castillo, Norma Cipatli Ayuzo Del Valle, Noemí García

**Affiliations:** 1 Escuela de Medicina y Ciencias de la Salud, Tecnologico de Monterrey, Monterrey Nuevo León, México; 2 The Institute for Obesity Research, Tecnológico de Monterrey, Monterrey Nuevo León, México; 3 Centro de Primera Infancia, Tecnológico de Monterrey, Monterrey Nuevo León, México

**Keywords:** circulating mtDNA, inflammation, mitochondrial DNA damage, obesity, oxidative stress

## Abstract

**Background:**

Childhood obesity is associated with cardiometabolic dysfunction. Oxidative stress and mitochondrial damage may contribute to this process, but their role children with excess adiposity and preserved metabolic parameters remains unclear. Circulating mitochondrial DNA (c-mtDNA) has emerged as a potential biomarker of mitochondrial injury and systemic stress. This study evaluated oxidative DNA damage and c-mtDNA integrity (c-mtDNAi) in children at different obesity phenotypes as indicators of metabolic risk.

**Methods:**

A total of 103 children aged 6–12 years were classified as normal-weight controls, preclinical obesity (pOb), or clinical obesity (cOb) using the 2025-OCF criteria based on biochemical alterations. Oxidative circulating DNA damage was quantified using 8-hydroxy-2′-deoxyguanosine (8-OH-dG). The c-mtDNAi was evaluated by long-range PCR. Lipid metabolism markers were measured, including triglycerides (TG), high-density lipoprotein cholesterol (HDL-C), the triglyceride-to-HDL cholesterol (TG/HDL-C) ratio, and the triglyceride–glucose (TyG) index. Plasma cytokine levels were quantified using flow cytometry.

**Results:**

Both preclinical Ob and clinical Ob groups showed significantly higher 8-OH-dG levels and reduced c-mtDNAi compared with controls (*p* < 0.0001), indicating mtDNA damage. 8-OH-dG was positively correlated with TG levels, the TG/HDL-C ratio, and the TyG index, whereas c-mtDNAi exhibited inverse correlations with these lipid-related markers and correlated positively with HDL-C levels. In multivariable analyses, waist circumference percentile remained independently associated with both 8-OH-dG and c-mtDNAi after adjustment for TG and HDL-C. Cytokine concentrations were elevated in pOb and cOb groups compared with controls.

**Conclusion:**

Oxidative DNA damage and reduced c-mtDNAi were detected in both pOb and cOb compared with controls, indicating mitochondrial and oxidative alterations associated with excess adiposity. These findings indicate that c-mtDNA integrity and 8-OH-dG may represent biomarkers associated with excess adiposity and metabolic risk in pediatric obesity.

**Clinical trial registration:**

ClinicalTrials.gov, TRN: NCT02320110, Registration date: 02 December 2014.

## 1 Introduction

Childhood obesity is one of the most significant public health challenges worldwide (1). In Mexico, the combined prevalence of overweight and obesity has reached 34.2% in school-aged children and 38.1% in adolescents according to ENSANUT 2023 (2). Excess adiposity is strongly associated with an increased risk of cardiometabolic disorders, including dyslipidemia, insulin resistance, type 2 diabetes, and cardiovascular disease (CVD) (3). Importantly, the biological processes that drive these complications may begin during childhood, long before the clinical appearance of conventional biochemical abnormalities (4–6).

Obesity is characterized by chronic metabolic stress, largely driven by lipid overload and mitochondrial dysfunction (7). Excess nutrient availability promotes mitochondrial overproduction of reactive oxygen species (ROS) (8, 9), leading to oxidative damage of cellular macromolecules, including DNA. Mitochondrial DNA (mtDNA) is particularly susceptible to oxidative injury due to its proximity to the electron transport chain, limited repair capacity, and lack of protective histones (10). Accumulating mtDNA damage can impair mitochondrial function, exacerbate oxidative stress (OxS), and contribute to metabolic dysregulation (11, 12).

Circulating cell-free DNA, including circulating nuclear DNA (c-nDNA) and circulating mitochondrial DNA (c-mtDNA), has emerged as a minimally invasive marker of cellular stress and tissue injury. In adults, alterations in circulating mtDNA levels and integrity have been associated with obesity (13–16), insulin resistance (17), systemic inflammation (18–20), and cardiometabolic risk (21). In addition, oxidized or fragmented mtDNA released into circulation may act as a danger-associated molecular pattern, activating innate immune pathways and sustaining low-grade inflammation (22). However, whether these mitochondrial and oxidative DNA alterations are already present in children classified with preclinical obesity remains insufficiently explored.

Recently, the concept of preclinical obesity (pOb) has been proposed to describe individuals with excess adiposity but preserved organ function and no detectable metabolic abnormalities (23). This framework enables the identification of patients who may exhibit biological alterations despite having biochemical parameters within reference ranges. Defining molecular changes associated with this phenotype could improve risk stratification and help identify children who may benefit from preventive interventions (24).

Although chronic low-grade inflammation has been described in pediatric obesity (5), evidence regarding oxidative DNA damage and c-mtDNA integrity (c-mtDNAi) in children, particularly within the pOb phenotype, is limited. Moreover, whether these molecular alterations are associated with metabolic stress and the way they relate to lipid-based cardiometabolic risk markers in childhood remains to be elucidated.

Therefore, this study aimed to determine whether children with pOb exhibit molecular and inflammatory alterations despite the absence of detectable metabolic abnormalities. Specifically, we evaluated oxidative damage to circulating DNA (c-DNA), c-mtDNAi, and systemic inflammatory cytokine profiles in Mexican children classified as healthy controls, pOb, or clinical obesity (cOb). In addition, we examined their associations with lipid-derived cardiometabolic risk indices.

## 2 Methods

This retrospective, cross-sectional study included 103 Mexican children aged 6–12 years. The study was conducted as a secondary analysis of a previously established pediatric cohort comprising approximately 1,300 school-aged children recruited in 2014 (25). For the present analysis, children were classified according to the Obesity Classification Framework (OCF 2025), and only those meeting all classification criteria with complete anthropometric, biochemical, and molecular data were included, resulting in a final analytical sample of 103 participants 29 controls, 18 preclinical obesity (pOb), and 56 clinical obesity (cOb).

Because this was a secondary analysis, the final sample size was determined by participant eligibility and data availability. To assess the adequacy of the analytical sample, a *post hoc* sample size analysis was performed using c-mtDNAi, the primary outcome of the study. Based on the observed effect size (f = 0.929), a minimum of 15 participants (5 per group) would have been required to achieve 80% power at *α* = 0.05 in a one-way ANOVA design.

The OCF 2025 integrates anthropometric measures of body size and adiposity with biochemical indicators of metabolic alteration. Body mass index (BMI) percentile (BMIp), calculated according to CDC growth charts, was used as a measure of body size, whereas waist circumference percentile (WCp) was included to better reflect central adiposity. Laboratory parameters were used to identify the presence of metabolic alterations. Participants were classified into three groups based on this framework:

- Control group: BMIp ≥ 5th and <85th percentile, with WCp within the reference range and normal biochemical parameters.
- Preclinical obesity (pOb): Elevated adiposity, defined as BMIp ≥ 85th percentile and WCp ≥ 90th percentile, with biochemical parameters within the reference range.
- Clinical obesity (cOb): Obesity, defined as BMIp ≥ 95th percentile, accompanied by at least one altered biochemical marker: high-density lipoprotein cholesterol (HDL-C), triglycerides (TG), or fasting glucose (see Section 2.2).

This approach allows the identification of children with increased adiposity before the development of metabolic abnormalities, as well as those with established obesity and metabolic alterations.

The exclusion criteria were as follows: medical contraindications indicated by the primary care physician, diagnosed inflammatory disorders (e.g., juvenile arthritis, Crohn's disease), and infectious diseases within the previous three weeks.

All procedures adhered to the Declaration of Helsinki and international ethical standards. The Ethics and Research Committees of the School of Medicine at Tecnologico de Monterrey (AIEMPPDM2SM), the Mexican Secretariat of Health (CONBIOETICA-19-CEI-011-20161017), and the Institutional Ethics Committee (13CI19039138) approved the protocol. Written informed consent was obtained from the patients’ parents or legal guardians.

### 2.1 Anthropometric and biochemical measurements

The anthropometric and biochemical parameters were analyzed, as previously described (26). The body weight, height, WCp, systolic blood pressure, and diastolic blood pressure were measured in each child. Data are presented as median and interquartile range for nonparametric variables and as the mean ± SD for parametric variables. Participants fasted for 12 h overnight before collecting blood samples. Five milliliters of blood were obtained under standard sterility conditions, collected in a 6-mL sterile BD vacutainer EDTA (10.8 mg) tube, and gently mixed by inversion. The tubes were then centrifuged at 1,000  ×   *g* for 20 min at 4 °C in a Beckman centrifuge using low acceleration and deceleration settings. Immediately after centrifugation, 1.5–2 mL of plasma were removed using a sterile pipette tip and aliquoted (0.1 mL) into sterile microtubes; one of the aliquots was used for DNA isolation, and the remaining aliquots were stored at −80 °C. The biochemical analysis included HDL-C, TG, and fasting glucose. Plasma TG, glucose, and HDL-C concentrations were measured by spectrophotometry with clinical-grade reagents from Pointe Scientific (Canton, MI, USA). Altered laboratory parameters were defined as: Fasting glucose > 100 mg/dL; HDL cholesterol < 40 mg/dL; TG > 100 mg/dL (<10 years) or >130 mg/dL (≥10 years) (24). Insulin resistance was estimated using two validated non–insulin-based indices, the triglyceride–glucose (TyG) index and the triglyceride-to-HDL cholesterol (TG/HDL-C) ratio, because fasting insulin concentrations were not available for all participants. The TyG index was calculated as: $TyG\;index=\ln\left(\frac{\;fasting\;triglycerides\;\left(\frac{mg}{dL}\right)\;\times \;fasting\;glucose\;\left(\frac{mg}{dL}\right)}{2}\right)$, and the TG/HDL-C ratio was obtained by dividing fasting TG by HDL-C (both in mg/dL). These indices have been proposed as reliable surrogate markers of insulin resistance and cardiometabolic risk in pediatric and adult populations (27–29).

### 2.2 Circulating DNA isolation

Immediately after plasma isolation, an aliquot of 90 µL was mixed with an equal volume of sterile phosphate-buffered saline (PBS) and centrifuged at 5,000  ×   *g* for 20 min to remove any debris and circulating cells. The PBS solution contained (in mM): 137 NaCl, 2.7 KCI, 8 Na_2_HPO_4_, and 2 KH_2_PO_4_, pH 7.4. Subsequently, the c-DNA was isolated from plasma using the QIAmp® DNA blood mini kit (Qiagen®, 51,106) as follows: 10 µg of proteinase K and 200 µL of AL buffer (Qiagen®) were added to plasma and incubated at 56 °C for 15 min. After that, c-DNA was isolated following manual instructions. Finally, c-DNA was quantified at 260/280 nm and stored at −80 °C. The isolated c-DNA was considered acceptable for analysis when the 260/280 nm ratio was encountered between 1.8 and 2.0.

### 2.3 c-DNA oxidation analysis

Oxidation of c-DNA was analyzed using the 8-hydroxy-2′-deoxyguanosine (8-OH-dG) ELISA kit (Abcam, ab201734). Briefly, 25 µL of c-DNA was incubated with 0.6 U of P1 nuclease at 37 °C for 15 min, followed by incubation with the primary antibody at 4 °C for 1 h. Thereafter, the plate was washed with PBS containing 0.05% Tween-20, followed by the addition of 100 µL of 3,3′,5,5′-Tetramethylbenzidine. The plate was then incubated for 30 min. Finally, the reaction was stopped using 0.16 M sulfuric acid, and the absorbance was measured at 450 nm. The concentration of 8-OH-dG was determined using the amount of c-DNA per µL, a standard curve and expressed in pg per 10 µg of DNA.

### 2.4 Long polymerase chain reaction endpoint

c-mtDNAi was analyzed using the long-range polymerase chain reaction endpoint (Long-PCR). A long mtDNA segment of ∼9,000 bp was amplified according to the kit manufacturer's instructions (GoTaqLong PCR Master Mix, Promega M4021), with the addition of 200 pmol of primers (Fw: 5′-AGCGCAAGTACCCACGTAAA-3 and Rv: 5′-TGGATAAGTGGCGTTGGCTT–3) and 30 ng of c-DNA, as previously reported by Vela-Guajardo et al. (30). Amplification was performed in a thermal cycler (Applied Biosystems® Veriti® 96-Well Fast Thermal Cycler, Waltham, MA, USA) with a single denaturation step (2 min at 95 °C), followed by 25 cycles of denaturation (15 s at 92 °C), and alignment/elongation (10 min at 65 °C), concluding with a final elongation step at 72 °C for 10 min. The amplicon was verified on a 1% agarose gel (image not shown) and quantified using PicoGreen and a standard curve (1–25 pg). For quantification, a 100 µL mixture containing 70 µL of Tris-EDTA buffer (10 mM Tris-HCl, 0.1 mM EDTA, pH 8.0), 5 µL of the amplicon, and 12.5 µM PicoGreen was incubated for 10 min and read at 485_ex_/528_em_.

c-mtDNA integrity was interpreted based on the relative abundance of the long mtDNA fragment because intact mtDNA is more likely to amplify as a long product, whereas fragmented/damaged mtDNA amplifies less efficiently. Therefore, higher long-fragment yield (relative to short-fragment DNA, when applicable) indicates greater mtDNA integrity, whereas lower yield reflects increased mtDNA fragmentation (30). PicoGreen-based quantification was validated by densitometric analysis of the gel bands (data not shown).

### 2.5 Mitochondrial DNA quantification by quantitative polymerase chain reaction

Previously, we found that the *MTND3* gene escapes from the mitochondria through the permeability transition pore after an increase in OxS (31). Therefore, a fragment of 221 bp from this gene was amplified to measure circulating mtDNA levels in plasma using the following primers: Fw: 5′-TGACTACCACAACTCAACGGCT-3′ and Rv: 5′-GCCAGACTTAGGGCTAGGATGA-3′. Nuclear DNA was quantified by amplifying a segment of the *β*-globin gene, using the following primers: Fw: 5′-AGTGCTCGGTGCCTTTAGTG-3′ and Rv: 5′-ATCAAGGGTCCCATAGACTCA-3′. Both genes were amplified using Brilliant III Ultra-Fast SYBR®Green (Agilent, 600,892) as follows: 1  ×   PCR reaction buffer, 200 nM of each primer, and 30 ng of c-DNA for each amplification reaction. Amplification was performed in a thermocycler (Stratagene Mx3005P; Agilent Technologies, Santa Clara, CA, USA) with a single denaturation step (5 min at 95 °C), followed by 40 cycles of 30 s of denaturation at 95 °C, 30 s of annealing at 55 °C, and 30 s of elongation at 72 °C. Quantification was performed using a standard curve for each gene. Subsequently, the amplicon corresponding to *MTND3* was sequenced to verify that it did not correspond to nuclear NUMTs (mitochondrial pseudogenes in nuclear chromosomes), resulting in 97% identity with the mitochondrial genome (see Supplementary Figure S1).

### 2.6 Plasma cytokine measurement

The cytokine profile was analyzed in plasma samples using the LEGENDplex™ Human Inflammation panel 1 for a multianalyte cytometric assay (BioLegend, San Diego, CA, USA). The panel included interleukin (IL)-1β, interferon (IFN)-*α*2, IFN-*γ*, tumor necrosis factor (TNF)-*α*, IL-6, CXCL8 (IL-8), IL-10, IL-12p70, IL-17A, IL-18, IL-23, and IL-33. Data were collected using a FACS-Canto II flow cytometer (Becton Dickinson, Franklin Lakes, NJ, USA). Each experiment was performed in triplicate, and the standard curve and LEGENDplex™ software (BioLegend) were used to calculate cytokine concentrations.

### 2.7 Statistical analysis

Statistical analyses were performed with Microsoft Excel®, R®, and GraphPad Prism®. Shapiro–Wilk tests were applied to each measured variable to evaluate the normality of the sample distribution. Differences among groups were assessed using one-way ANOVA followed by Bonferroni's multiple comparisons test for normally distributed variables, or Kruskal–Wallis followed by Dunn's multiple comparisons test for nonparametric variables. Age distribution was compared among groups using the Kruskal–Wallis test with Dunn's multiple comparisons. Sex distribution was evaluated using Fisher's exact test. Pearson's (parametric) or Spearman's (nonparametric) correlation coefficients were used to assess associations between c-mtDNAi, 8-OH-dG, and metabolic variables. Multiple linear regression analyses were performed using c-mtDNAi or 8-OH-dG as dependent variables and WC percentile, TG, and HDL-C as independent variables. Statistical significance was established at *p* < 0.05.

## 3 Results

### 3.1 Patients’ medical reports and clinical characteristics

A total of 103 children aged 6–12 years were included in this study, with a balanced sex distribution (53 girls and 50 boys). The median age was 8.0 (7.0–10.0) years in controls, 8.5 (7.0–10.25) years in pOb, and 9.0 (7.25–10.0) years in cOb. Age distribution did not differ significantly among groups (Kruskal–Wallis statistic = 0.953, *p* = 0.621). Similarly, sex distribution was comparable among groups (Fisher's exact test, *p* = 0.226). Anthropometric characteristics of the study population are shown in Table 1. Waist circumference was included to complement the assessment of central fat accumulation, which is more closely associated with cardiometabolic risk, because BMI alone does not reflect fat distribution (23, 24). Based on BMI-for-age and WCps, 29 participants (28.15%) were classified as healthy controls, and 74 participants were included in the obesity group (71.85%). The obesity group was further divided into preclinical (*n* = 18) and clinical (*n* = 56) obesity groups based on biochemical markers, allowing the identification of children with elevated adiposity with or without metabolic alterations.

**Table 1:** Clinical characteristics of the study population.

Variable	Control (*n* = 29)	pOb (*n* = 18)	cOb (*n* = 56)	*P value*	*P value*	*P value*
				Control vs. pOb	Control vs. cOb	pOb vs. cOb
Male/Female	12/17	12/6	26/30			
Age (years)	8.0 (7.0–10.0)	8.5 (7.0–10.25)	9.0 (7.25–10.0)	>0.9999	0.9871	>0.9999
Waist circumference (percentile)	25.0 (10.0–25.0)	93.0 (85.8–98.7)	94.8 (91.4–98.9)	<0.0001	<0.0001	0.6262
BMI (percentile)	28.0 (21.0–43.5)	97.6 (97.0–99.1)	98.8 (97.8–99.3)	<0.0001	<0.0001	0.8284
Labs						
TG (mg/dL)	81 (64.2–89.2)	95.0 (79.5–118.5)	157.0 (113.8–188.8)	0.1383	<0.0001	0.0010
HDL-C (mg/dL)	46.0 (43.0–47.0)	45.0 (37.0–54.2)	34.5 (31.2–38.7)	0.9127	<0.0001	<0.0001
Glucose (mg/dL)	78.0 (70.5–82.0)	83.0 (80.5–87.2)	82.0 (77.0–90.0)	0.0424	0.0452	>0.9999
Clinical factors						
Systolic BP (percentile)	ND	69.0 (56.5–81.2)	75.0 (58.2–89.5)	ND	ND	0.2769
Diastolic BP (percentile)	ND	45.2 ± 16.1	47.9 ± 19.4	ND	ND	0.5709
Associated factors						
TyG index	8.05 (7.7–8.1)	8.2 (8.0–8.5)	8.7 (8.4–8.9)	0.1110	<0.0001	0.0031
TG/HDL-C ratio	1.7 (1.3–1.9)	2.0 (1.5–2.9)	4.5 (2.9–6.1)	0.3254	<0.0001	<0.0001

Data are presented as median and interquartile range for nonparametric data and as mean ± SD: for parametric data. ND, no data; BP, blood pressure; pOb, preclinical obesity; cOb, clinical obesity; TG, triglycerides; HDL-C, high-density lipoprotein cholesterol; BMI, body mass index. Age comparisons were performed using the Kruskal–Wallis/Dunn (overall *p* = 0.621). Sex distribution was compared using Fisher's exact test (overall *p* = 0.226). Anthropometric and biochemical variables were performed with ANOVA/Bonferroni or Kruskal–Wallis/Dunn, as described in methods.

As expected, children with cOb exhibited a worsened metabolic profile compared with those in the controls and with pOb. TG levels, the TyG index, and the TG/HDL-C ratio were significantly higher in the cOb group, whereas HDL-C levels were markedly lower (*p* < 0.0001 vs. controls). This approach allowed them to be distinguished from the pOb group. Glucose levels were elevated in both pOb and cOb compared with controls (*p* = 0.0424 and *p* = 0.0452, respectively), although they did not differ between obesity groups. The waist circumference and BMI percentiles were significantly higher in the pOb and cOb groups than in the controls (*p* < 0.0001).

### 3.2 Cytokine alterations in children with obesity

Obesity causes chronic low-grade inflammation, mainly shown in studies with adults (32). In our study, plasma cytokine concentrations were quantified to evaluate inflammation. As shown in Table 2, several cytokines were already elevated in the pOb group compared with controls, including IL-6, IL-8, IL-17, IL-23, and IL-33, and remained similarly high in the cOb group, with the exception of IL-6 and IL-8, which were significantly increased only in the preclinical phenotype. By contrast, IL-12, IFN-*γ* and TNF-α showed significant differences only in the cOb group than in the control group, indicating that these cytokines may be more closely associated with metabolic alterations observed in children classified as cOb, including elevated triglycerides, reduced HDL-C, and/or higher fasting glucose concentrations.

**Table 2:** Cytokine concentrations (pg/mL) in children with pOb and cOb.

Cytokine	Control (*n* = 29)	pOb (*n* = 18)	cOb (*n* = 56)	*p value* Control vs. pOb	*p value* Control vs. cOb	*p value* pOb vs. cOb
IL-1β	0.3 (0.3–1.5)	0.3 (0.3–2.4)	0.3 (0.3–2.8)	>0.9999	>0.9999	>0.9999
IL-6	1.0 (0.3–3.4)	4.2 (0.9–6.9)	2.7 (0.9–6.6)	0.0479	0.0510	>0.9999
IL-8	1.3 (0.6–2.6)	4.6 (1.8–8.4)	2.3 (0.6–6.7)	0.0268	0.3769	0.3272
IL-10	1.4 (0.6–2.7)	3.0 (1.2–5.2)	3.2 (0.9–5.1)	0.1207	0.0508	>0.9999
IL-12	1.0 (0.7–2.0)	2.3 (1.2–3.6)	3.0 (1.0–4.3)	0.0875	0.0028	>0.9999
IL-17	0.3 (0.3–0.3)	4.9 (0.4–17.3)	4.2 (0.6–13.2)	0.0001	<0.0001	>0.9999
IL-18	83.4 (48.0–159.2)	121.4 (75.6–177.1)	107.1 (4.2–198.5)	0.7955	>0.9999	>0.9999
IL-23	3.0 (1.3–5.0)	7.6 (5.1–15.4)	9.2 (3.7–18.2)	0.0035	0.0002	>0.9999
IL-33	3.6 (3.6–19.1)	57.0 (17.7–160.0)	99.4 (43.1–167.9)	0.0004	<0.0001	0.8060
IFN-α	6.5 (3.3–13.3)	5.0 (0.2–28.7)	7.2 (3.0–23.0)	>0.9999	>0.9999	>0.9999
IFN-*γ*	13.1 (0.4–19.3)	20.4 (6.2–32.2)	20.4 (9.8–40.9)	0.3472	0.0045	>0.9999
TNF-α	0.7 (0.7–0.7)	3.1 (0.7–13.8)	4.3 (0.7–13.9)	0.1435	0.0093	>0.9999

Data are presented as median and interquartile range. Tests were performed with Kruskal–Wallis/Dunn as described in Section 2. pOb, preclinical obesity; cOb, clinical obesity; TNF, tumor necrosis factor; IFN, interferon; IL, interleukin.

Notably, cytokine levels did not further increase in the cOb group compared with the pOb group, despite the presence of altered biochemical parameters in cOb. This result suggest that systemic inflammation is present in children with excess adiposity even in the absence of detectable metabolic abnormalities and remains elevated in children classified as cOb. Overall, these findings support inflammation as a sustained feature of excess adiposity in childhood.

### 3.3 Oxidative DNA damage and mtDNA integrity in obesity

**Figure 1:**
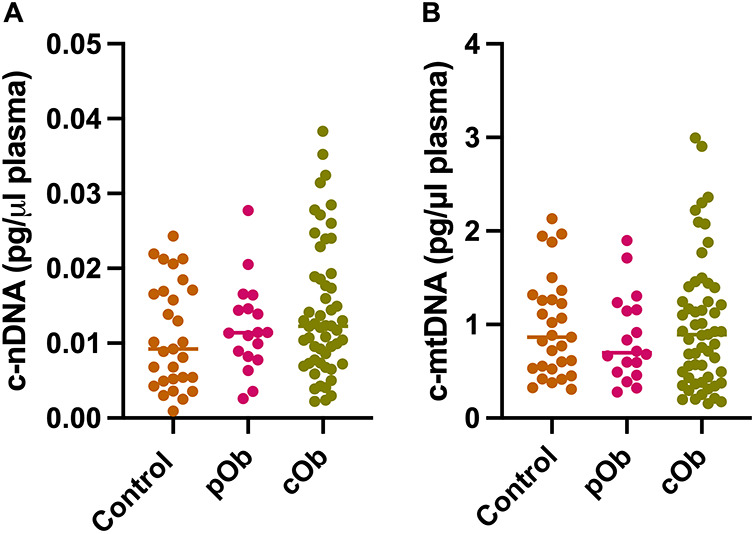
Analysis of c-nDNA and c-mtDNA levels in children with obesity. **(A)** c-nDNA analyzed as *β*-globin. **(B)** quantification of *MTND3*. Groups: control (orange, *n* = 29), preclinical obesity (pOb; pink, *n* = 18), and clinical obesity (cOb; green, *n* = 56). Tests were performed with ANOVA and Kruskal–Wallis with Dunn's test. *p < 0.05, **p < 0.01, ***p < 0.001, and ****p < 0.0001. circulating nuclear DNA (c-nDNA); circulating mitochondrial DNA: c-mtDNA; pOb: preclinical Obesity; cOb: clinical Obesity. Figure 1 Dot plots showing individual circulating nuclear DNA (*β*-globin) and mitochondrial DNA (*MTND3*) levels in control, preclinical obesity, and clinical obesity groups of children. Mean values with variability bars are displayed for each group, with statistically significant differences indicated by asterisks.

We analyzed the oxidation of c-DNA and c-mtDNA integrity as indicators of OxS and mitochondrial injury to investigate the relationship between obesity and c-mtDNA alterations. As a first step, circulating nuclear DNA (c-nDNA) and c-mtDNA were quantified to assess the presence of both DNAs in the plasma. As shown in Figures 1A,B, c-nDNA and c-mtDNA levels did not differ significantly among the groups. These results indicate that the total amount of DNA into circulation does not differ between pOb and cOb, suggesting that differences associated with excess adiposity may be related to qualitative changes in DNA integrity rather than increased amounts of c-mtDNA.

**Figure 2:**
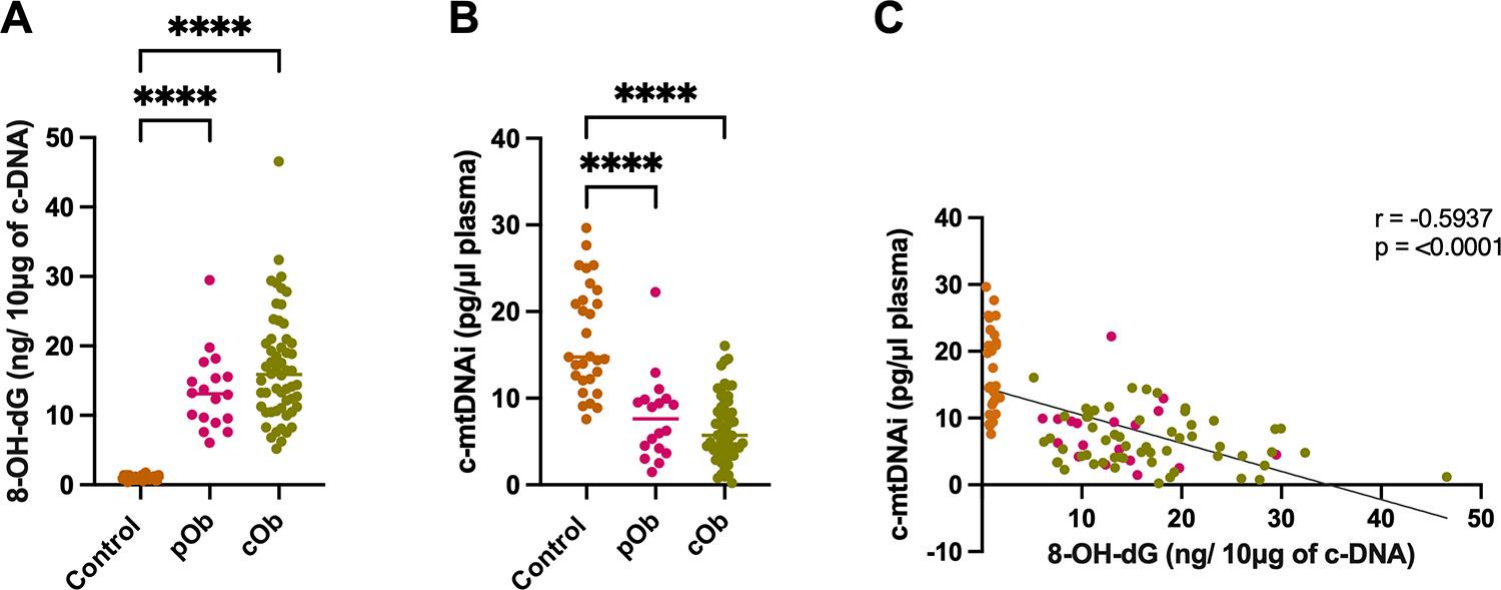
Analysis of c-DNA oxidation and c-mtDNA integrity in children with obesity. **(A)** Plasma levels of 8-OH-dG. **(B)** c-mtDNAi quantified by Long-PCR. **(C)** Correlation between c-mtDNAi and 8-OH-dG. Groups: control (orange, *n* = 29), preclinical obesity (pOb; pink, *n* = 18), and clinical obesity (cOb; green, *n* = 56). Tests were performed with ANOVA and Kruskal–Wallis with Dunn's test. *p < 0.05, **p < 0.01, ***p < 0.001, and ****p < 0.0001. Circulating oxidized DNA: 8-OH-dG, 8-hydroxy-2-deoxyguanosine; circulating intact mitochondrial DNA, c-mtDNAi; pOb, preclinical obesity; cOb, clinical obesity. Figure 2 Panels showing plasma 8-hydroxy-2-deoxyguanosine levels, circulating intact mitochondrial DNA quantified by Long-PCR, and their correlation in children across control, preclinical obesity, and clinical obesity groups. Group comparisons and a scatter plot with regression line illustrate the relationship between oxidative DNA damage and mitochondrial DNA integrity.

Oxidative damage to c-DNA was evaluated by quantifying 8-OH-dG levels in c-DNA extracts from all groups. As shown in Figure 2A, the level of 8-OH-dG was significantly higher in the pOb (*p* < 0.0001) and cOb (*p* < 0.0001) groups compared with the control group. These results may reflect that oxidative damage to c-DNA is already present in children with excess adiposity, identified by a waist circumference above the 95th percentile, and remains elevated in children classified as cOb.

In addition, c-mtDNAi was evaluated using Long-PCR. The c-mtDNAi was significantly reduced in the pOb and cOb groups compared with that in the control group (*p* < 0.0001 for both comparisons) (Figure 2B). These data may indicate that mitochondrial genome injury is present in children classified as pOb and is also evident in children classified as cOb. Then, a correlational analysis was conducted between c-mtDNAi and 8-OH-dG results, showing a moderate, significant negative correlation with 8-OH-dG levels (Figure 2C). The results indicate that the increased OxS could be related to the reduction of c-mtDNAi.

### 3.4 Associations between oxidative and mitochondrial DNA integrity and metabolic risk markers

**Figure 3:**
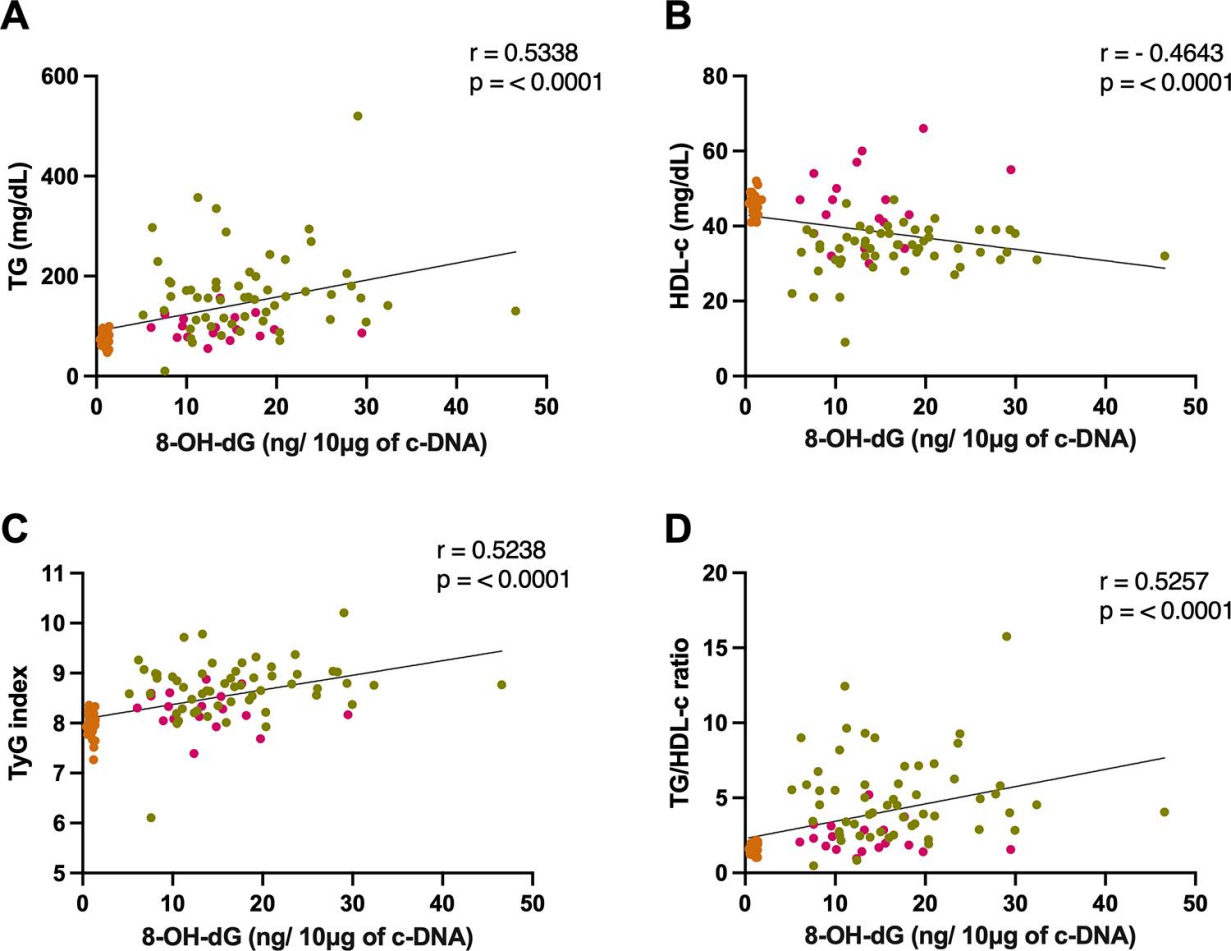
Correlation between oxidative stress markers and metabolic risk parameters. Scatter plots show the relationship between 8-hydroxy-2′-deoxyguanosine (8-OH-dG) levels and **(A)** TG levels, **(B)** HDL-C levels, **(C)** the TyG index, and **(D)** the TG/HDL-C ratio in all study participants. Each point represents an individual subject: control (orange, *n* = 29), preclinical obesity (pOb; pink, *n* = 18), and clinical obesity (cOb; green, *n* = 56). Correlations were determined using Spearman's rank test for nonparametric data and Pearson's test for parametric data. The line represents the linear regression fit. Circulating oxidized DNA: 8-OH-dG, 8-hydroxy-2-deoxyguanosine; pOb, preclinical obesity; cOb, clinical obesity. Figure 3 Scatter plots showing associations between circulating 8-hydroxy-2-deoxyguanosine levels and triglycerides, HDL cholesterol, TyG index, and triglyceride-to-HDL ratio in all participants. Each point represents an individual child from control, preclinical obesity, or clinical obesity groups, with linear regression lines displayed.

Correlation analyses were conducted between 8-OH-dG and biochemical parameters across the study cohort to investigate whether OxS is linked metabolic alterations and cardiometabolic risk markers. As shown in Figure 3, 8-OH-dG levels were positively correlated with TG levels (r = 0.5338, *p* = <0.0001) (Figure 3A) and lipid-based cardiometabolic risk indices, including the TG/HDL-C ratio (r = 0.5257, *p* = <0.0001) (Figure 3C) and the TyG index (r = 0.5238, *p* = <0.0001) (Figure 3D). In addition, a modest but significant inverse correlation was observed with HDL-C levels (r = −0.4643, *p* = <0.0001) (Figure 3B), suggesting greater oxidative DNA damage in children with a more unfavorable lipid profile. No significant association was detected between 8-OH-dG and glucose levels.

**Figure 4:**
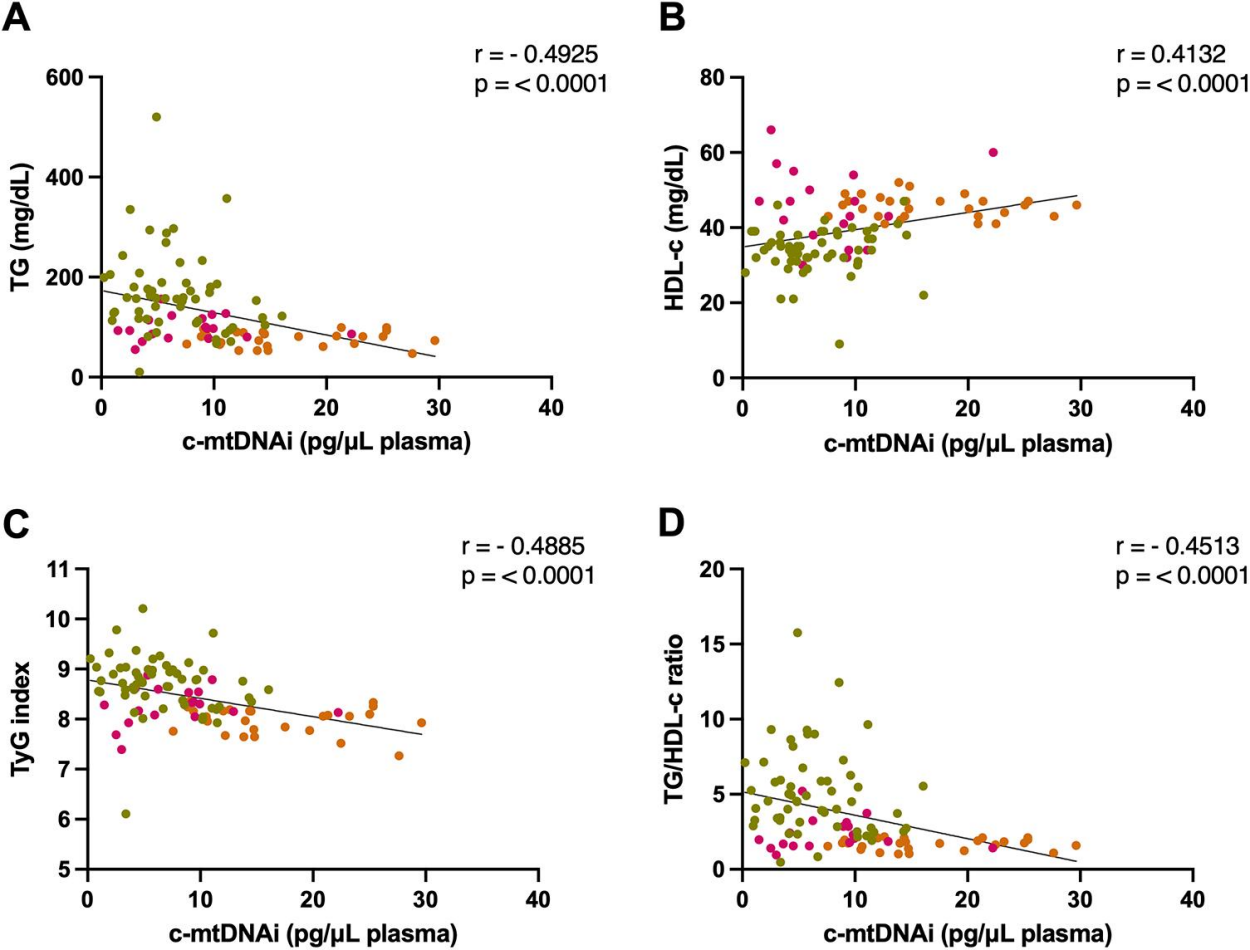
Correlation between mitochondrial DNA integrity and metabolic risk parameters. Scatter plots show the relationship between mtDNA integrity (c-mtDNAi) levels and **(A)** TG levels, **(B)** HDL-C levels, **(C)** the TyG index, and **(D)** the TG/HDL-C ratio in all study participants. Each point represents an individual subject: control (orange, *n* = 29), preclinical obesity (pOb; pink, *n* = 18), and clinical obesity (cOb; green, *n* = 56). Correlations were determined using Spearman's rank test for nonparametric data and Pearson's test for parametric data. The line represents the linear regression fit. Circulating oxidized DNA: 8-OH-dG, 8-hydroxy-2-deoxyguanosine; pOb, preclinical obesity; cOb, clinical obesity. Figure 4 Scatter plots showing relationships between circulating mitochondrial DNA integrity and triglycerides, HDL cholesterol, TyG index, and triglyceride-to-HDL ratio in children from control, preclinical obesity, and clinical obesity groups. Individual data points and linear regression fits are shown.

Similarly, c-mtDNA integrity was evaluated in relation to biochemical markers of metabolic risk. As shown in Figure 4, c-mtDNAi was significantly negatively correlated with TG levels (r = −0.4925, *p* = <0.0001), as well as with lipid-derived risk indices, including the TyG index (−0.4885, *p* = <0.0001) and the TG/HDL-C ratio (r = −0.4513, *p* = <0.0001). By contrast, a moderate and significant positive correlation was observed with HDL-C levels (r = 0.4132, *p* = <0.0001). These results suggest that lower c-mtDNA integrity may be linked to a more unfavorable lipid profile. Overall, these findings support the possibility that mitochondrial genome damage may be closely associated with alterations in lipid metabolism and cardiometabolic risk markers in children with excess adiposity.

Additionally, to further evaluate the independent associations of anthropometric and metabolic variables with c-mtDNAi and oxidative DNA damage, multiple linear regression analyses were performed using WCp, TG, and HDL-C as predictors (Supplementary Tables S1 and S2). For c-mtDNAi, the overall model was significant (R^2^ = 0.428, adjusted R^2^ = 0.410, *p* < 0.0001). WCp was significantly associated with c-mtDNAi (*β* = −0.119, 95% CI: −0.154 to −0.084, *p* < 0.0001), whereas TG and HDL-C were not significant predictors in the multivariate model.

Similarly, the regression model for 8-OH-dG was significant (R^2^ = 0.482, adjusted R^2^ = 0.466, *p* < 0.0001). WCp was significantly associated with 8-OH-dG levels (*β* = 0.177, 95% CI: 0.130 to 0.224, *p* < 0.0001). Although TG showed a positive trend, it did not reach statistical significance (*p* = 0.060), and HDL-C was not a significant predictor (*p* = 0.729).

## 4 Discussion

In this study, we show that children with excess adiposity present alterations related to mitochondrial stress and oxidative imbalance, including those classified as pOb. We identified a subgroup of children with pOb, defined as an obesity phenotype with increased adiposity but preserved organ function and biochemical markers within reference ranges (24), by applying the 2025-OCF classification (23). This classification enabled us to identify alterations in mtDNA integrity, oxidative DNA damage, and inflammation, highlighting biological differences between obesity phenotypes (33–35). An important consideration is that the OCF framework is relatively recent and that the present study does not establish a temporal progression from pOb to cOb. Therefore, our findings should not be interpreted as evidence that all children classified as pOb will necessarily develop cOb. Rather, this framework provides a useful approach for distinguishing children with excess adiposity but preserved metabolic parameters from those exhibiting metabolic abnormalities, enabling the identification of differences in oxidative stress, inflammation, and mitochondrial genome integrity.

Pediatric studies have reported that obesity is associated with increased oxidative DNA damage and genomic instability, which correlate with adiposity and inflammatory status and may predict future metabolic risk in children and adolescents (35). In addition, evidence in pediatric obesity indicates dysregulation of mitochondrial markers and redox balance, supporting the idea that mitochondrial dysfunction and OxS are linked to metabolic disturbances in obesity (36).

Our findings are consistent with the concept that excess adiposity may act as an important biological stressor associated with mitochondrial dysfunction and oxidative damage (37, 38). Experimental and clinical evidence indicates that increased mitochondrial ROS production in adipose tissue has been associated with obesity, before detectable impairments in mitochondrial respiratory capacity or the appearance of classical metabolic abnormalities (39, 40). Mechanistically, excess adipose tissue can generate ROS through adipocyte metabolic activity, mitochondrial dysfunction, and NADPH oxidase pathways, implicating adiposity as a key mediator of OxS (7).

mtDNA has emerged as a central mediator of metabolic stress. In adults with obesity and type 2 diabetes (T2D), c-mtDNA levels have been correlated with insulin resistance and systemic inflammation (41, 42). In our cohort, mtDNA damage and inflammation were already evident in children classified as pOb.

Although total c-mtDNA levels did not differ between the study groups, c-mtDNA integrity was significantly reduced in pOb and cOb children. This result suggests that mtDNA damage may occur by excess adiposity (43). Reduced mtDNA integrity may reflect a higher sensitivity to lipotoxicity and ROS overproduction, consistent with adult studies linking OxS to mtDNA fragmentation and release (15, 16, 44). Therefore, impaired c-mtDNA integrity may represent a molecular sign of metabolic stress that is detectable in children with excess adiposity despite having conventional biochemical parameters within reference ranges.

The observed associations between c-mtDNAi and lipid-related parameters may be biologically plausible given the susceptibility of mitochondrial DNA to oxidative injury (11). Mitochondrial DNA is located in close proximity to the electron transport chain, a major source of reactive oxygen species, and lacks the protective histone structures present in nuclear DNA (45). Consequently, persistent OxS may promote mtDNA damage and impair mitochondrial function. In turn, mitochondrial dysfunction may alter cellular energy metabolism and further increase OxS, potentially contributing to a cycle of metabolic dysregulation (46, 47). The negative correlations observed between c-mtDNAi and TG, TyG, and TG/HDL-C ratio, together with the positive association with HDL-C, suggest that alterations in mitochondrial genome integrity may accompany an unfavorable metabolic profile in children with excess adiposity.

Similarly, 8-OH-dG was elevated in both obesity groups. This finding aligns with previous reports of increased OxS in children with excess adiposity (48), as well as in adult dyslipidemia, atherosclerosis, and T2D (49, 50). In our cohort, oxidative DNA damage showed the strongest associations with TG levels, HDL-C levels, and lipid-based indices, suggesting that lipid disturbances may be associated with OxS (43). By contrast, glucose levels were less relevant in the pOb phenotype.

An interesting finding was the presence of increased inflammatory markers in children classified as pOb despite the absence of significant differences in TyG index and TG/HDL-C ratio compared with controls. Although inflammation and insulin resistance are closely linked, these processes may not emerge simultaneously during obesity development (51). Adipose tissue expansion has been associated with activation of inflammatory pathways and cytokine production (52) that may occur before detectable alterations in surrogate markers of insulin resistance (53). In this context, the inflammatory profile observed in the pOb group may reflect a biological response to excess adiposity. Furthermore, because fasting insulin concentrations were not measured, subtle alterations in insulin sensitivity can not be excluded. The temporal relationship between inflammation and metabolic dysfunction remains to be clarified using direct assessments of insulin resistance.

Notably, WCp remained significantly associated with both c-mtDNAi and 8-OH-dG in multivariate analyses after accounting for TG and HDL-C. Although the cross-sectional design does not permit causal inference, these findings suggest that excess adiposity may be more closely associated with oxidative DNA damage and mitochondrial genome alterations than individual lipid parameters. This observation is consistent with the concept that adipose tissue expansion may contribute to oxidative and inflammatory alterations through multiple mechanisms that extend beyond circulating lipid abnormalities alone (52, 54, 55).

In our cohort, several pro-inflammatory cytokines (e.g., IL-17, IL-23, and IL-33) were elevated in pOb and cOb, supporting the concept that low-grade inflammation emerges with excess adiposity (54, 56, 57). Notably, IL-6 and IL-8 were significantly elevated only in children classified as pOb, which may reflect inflammatory signaling asociated with excess adiposity in the absence of detectable metabolic abnormalities (58–60). By contrast, IL-12, IFN-*γ*, and TNF-α showed significant differences only in the cOb group compared with the controls. This pattern may reflect immune and metabolic processes associated with the metabolic alterations observed in children classified as cOb (57, 61, 62). IL-12 and IFN-*γ* are associated with Th1 polarization and macrophage activation (63–66). Recent evidence highlights the IFN-*γ*–IL-12 axis in obesity-associated metabolic inflammation, where macrophage IFN-*γ* signaling promotes IL-12 production and sustains pro-inflammatory immune responses linked to metabolic dysfunction (67). TNF-α is a key mediator of immune activation that has been linked to adipose tissue inflammation and insulin signaling disruption in obesity (68). Together, these findings suggest that although many inflammatory markers increase with adiposity, changes in IL-12, IFN-*γ*, and TNF-α may be more closely associated with the metabolic abnormalities observed in children classified as cOb. Notably, these cytokines were not elevated in children classified as pOb despite the presence of increased adiposity and other inflammatory alterations, suggesting that distinct inflammatory pathways may be associated with different obesity phenotypes. However, these associations should be interpreted with caution because this study is observational and does not include mechanistic experiments. Additional mechanistic studies (e.g., *in vitro* models of adipocytes and immune cells or *ex vivo* stimulation assays) will be important to determine whether IL-12, IFN-*γ*, and TNF-α contribute to obesity-associated metabolic dysfunction or represent downstream markers of immune activation associated with metabolic alterations.

No significant differences in arterial blood pressure were observed among the study groups despite the presence of oxidative DNA damage, impaired mtDNA integrity, and inflammatory alterations. Although OxS and mitochondrial dysfunction have been implicated in the development of endothelial dysfunction and hypertension (69, 70), the absence of blood pressure differences in our cohort suggests that molecular alterations associated with excess adiposity may reflect biological disturbances that are detectable even in the absence of clinically measurable cardiovascular manifestations (53, 71, 72). Whether these alterations are associated with subsequent changes in blood pressure remains to be determined.

From a translational perspective, in addition to waist circumference, c-mtDNAi and oxidative DNA damage may represent promising biomarkers for metabolic risk in pediatric obesity. Our findings indicate that mitochondrial genome damage and OxS are detectable in children classified as pOb, highlighting their potential utility in identifying biological alterations associated with excess adiposity. The detection of these molecular alterations may offer a time for intervention before the development of clinically evident cardiometabolic complications.

The consistent pattern observed across mitochondrial, oxidative, and inflammatory markers indicates relevant biological effects despite the limited cohort size. Future longitudinal studies are needed to determine whether these biomarkers are associated with the subsequent development of obesity-related metabolic comorbidities, including insulin resistance, type 2 diabetes, and CVD, later in life. Defining the cellular origin of c-DNA and testing whether these alterations improve with lifestyle interventions will also be important for clinical translation.

## 5 Conclusion

In this study, we show that mtDNA alterations are already evident in children with excess adiposity before the onset of overt biochemical abnormalities. Although the circulating mtDNA quantity did not differ between groups, oxidative DNA damage and reduced mtDNA integrity were present in pOb and persisted in cOb. These findings suggest that mitochondrial genome instability and OxS may be present in children with excess adiposity.The identification of mtDNA damage may therefore provide a potential opportunity for timely lifestyle or clinical interventions aimed at reducing future cardiometabolic risk.

## 6 Limitations

This study has several limitations that should be considered when interpreting the findings. First, the cross-sectional design precludes conclusions regarding causality or the temporal evolution of the obesity phenotypes identified using the OCF framework. Consequently, it was not possible to determine whether children classified as pOb subsequently develop cOb or represent a distinct obesity phenotype, and longitudinal studies are required to establish the temporal stability, clinical reproducibility, and prognostic value of these phenotypes. Second, the relatively small number of children classified as pOb may have limited the ability to detect more subtle differences between groups. Third, oxidative mtDNA damage was assessed indirectly through c-mtDNA integrity measurements, and circulating mtDNA is subject to biological variability. Furthermore, the cellular origin of c-mtDNA could not be determined in the present study. In addition, fasting insulin concentrations were not available; therefore, insulin resistance could only be estimated using surrogate markers such as the TyG index and TG/HDL-C ratio. Dietary intake and pubertal status (Tanner staging), both of which may influence OxS, inflammatory responses, and metabolic parameters, were not assessed. Future studies incorporating longitudinal designs and mechanistic approaches will be necessary to further characterize the relationship between adiposity, mitochondrial dysfunction, inflammation, and metabolic health in pediatric populations.

## Data Availability

The raw data supporting the conclusions of this article will be made available by the authors, without undue reservation.
