# Supplementary material for: Reduced circulating mitochondrial DNA integrity and increased DNA oxidation in preclinical and clinical pediatric obesity: an observational study
Source: Front Pediatr. 2026 Jun 29;14:1813689. doi: 10.3389/fped.2026.1813689 (PMC13357151; doi:10.3389/fped.2026.1813689)
Supplement: Supplementary file 1 [file Supplementaryfile1.docx]

***Supplementary Information***

Early detection of circulating mitochondrial DNA damage in a small pediatric cohort with preclinical and clinical obesity

Mónica M. Velásquez-Esparza^1^, Perla Pérez-Treviño^2,3^, Leticia Elizondo-Montemayor^1^, Gerardo García-Rivas^2^, Elena González^1,2^, Cipatli Ayuzo^1,2^ and Noemí García*^1,2^


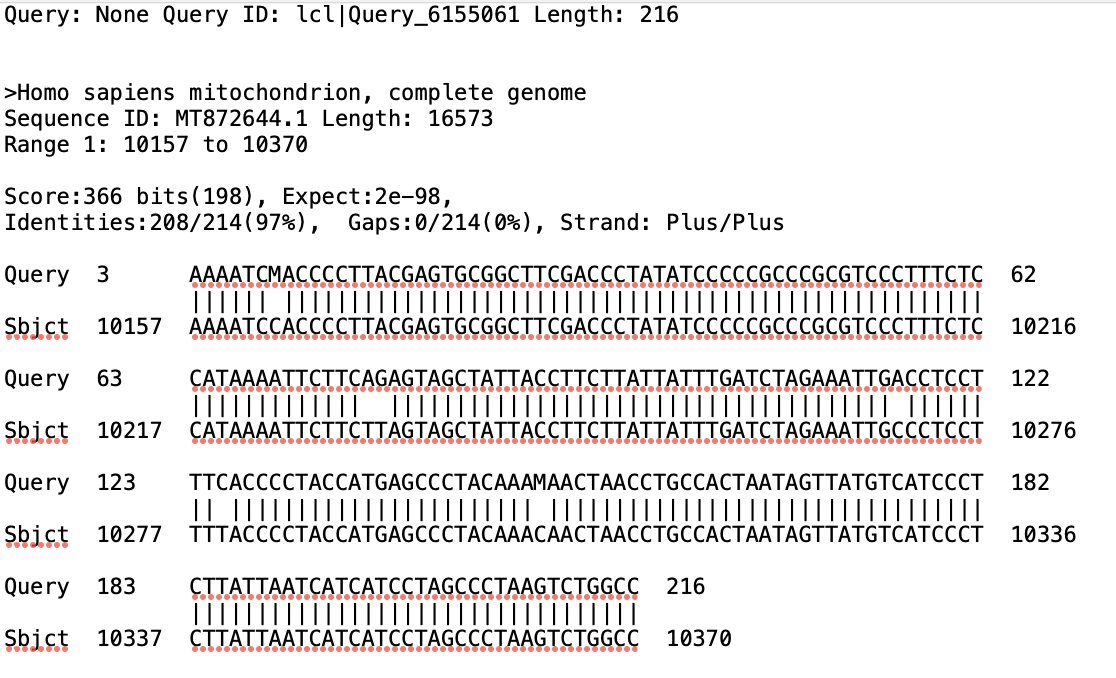


**Fig. S1**. MTND3 sequence alignment was obtained by Sanger sequence as follows: After MTDN3 amplification by PCR using as a template a sample of a patient, this was purified using Qiagen columns, quantified, and sent for sequencing. The sequence was analyzed on the NCBI website.

**Supplementary Table S1.** Multivariate linear regression model for c-mtDNAi

| Predictor | β coefficient | 95% CI | *p value* |
| --- | --- | --- | --- |
| WCp | -0.1191 | -0.1538 to -0.0844 | <0.0001 |
| TG | -0.0114 | -0.0260 to 0.0032 | 0.1239 |
| HDL-C | 0.0054 | -0.0548 to 0.0656 | 0.8582 |

Model statistics:

- R² = 0.428
- Adjusted R² = 0.410
- Overall p < 0.0001

Data are presented as regression coefficients (β), 95% confidence intervals (95% CI), and *p values*. The dependent variable was c-mtDNAi, circulating mitochondrial DNA integrity. Independent variables included WCp, waist circumference percentile; TG, triglycerides; and HDL-C, high-density lipoprotein cholesterol. Statistical significance was established at p < 0.05.

**Supplementary Table S2.** Multivariate linear regression model for 8-OH-dG

| Predictor | β coefficient | 95% CI | *p value* |
| --- | --- | --- | --- |
| WC percentile | 0.1767 | 0.1297 to 0.2236 | <0.0001 |
| TG | 0.0189 | -0.0008 to 0.0387 | 0.0604 |
| HDL-C | -0.0143 | -0.0958 to 0.0672 | 0.7287 |

Model statistics:

- R² = 0.482
- Adjusted R² = 0.466
- Overall p < 0.0001

Data are presented as regression coefficients (β), 95% confidence intervals (95% CI), and *p values.* The dependent variable was 8-OH-dG, 8-hydroxy-2′-deoxyguanosine. Independent variables included WCp, waist circumference percentile; TG, triglycerides; and HDL-C, high-density lipoprotein cholesterol. Statistical significance was established at p < 0.05
